# Total flavone of *Abelmoschus Manihot* improves colitis by promoting the growth of *Akkermansia* in mice

**DOI:** 10.1038/s41598-021-00070-7

**Published:** 2021-10-21

**Authors:** Fan Bu, Yang Ding, Tuo Chen, Qiong Wang, Rong Wang, Jin-yong Zhou, Feng Jiang, Dan Zhang, Minmin Xu, Guoping Shi, Yugen Chen

**Affiliations:** 1 Department of Colorectal Surgery, The Affiliated Hospital of Nanjing University of Chinese Medicine, 155 Hanzhong Road, Nanjing, 210029 Jiangsu China; 2 Nanjing University of Chinese Medicine, Nanjing, Jiangsu China; 3 General Surgery, Affiliated Hospital of Yangzhou University, Yangzhou, Jiangsu China; 4 Central Laboratory, Affiliated Hospital of Nanjing University of Chinese Medicine, Jiangsu Province Hospital of Chinese Medicine, NanjingJiangsu, 210029 China

**Keywords:** Ulcerative colitis, Clinical microbiology

## Abstract

The total flavone of *Abelmoschus manihot* (TFA), a compound extracted from the flowers of *Abelmoschus manihot* (L.) Medic, has been widely used for the treatment of Crohn's disease, chronic glomerulonephritis and other diseases. The aim of this study was to investigate the effect of TFA on the gut microbiota and intestinal barrier in dextran sulfate sodium (DSS)-induced experimental colitis. C57BL/6J mice were treated with 2.5% DSS in drinking water to induce colitis. Mice were orally administered TFA (62.5 mg/kg, 125 mg/kg) or prednisone acetate (PAT, 2.5 mg/kg) once daily for 7 days. Biological samples were collected for analysis of inflammatory cytokines, gut microbiota and intestinal barrier integrity. TFA-H (125 mg/kg) markedly attenuated DSS-induced colon shortening and histological injury in experimental colitis. The therapeutic effect was similar to that of PAT administration. TFA-H notably modulated the dysbiosis of gut microbiota induced by DSS and greatly enriched *Akkermansia muciniphila* (*A. muciniphila*). Moreover, TFA-H remarkably ameliorated the colonic inflammatory response and intestinal epithelial barrier dysfunction. Interestingly, TFA directly promotes the growth of *A. muciniphila *in vitro. Taken together, the results revealed for the first time that TFA, as a prebiotic of *A. muciniphila*, improved DSS-induced experimental colitis, at least partly by modulating the gut microflora profile to maintain colonic integrity and inhibit the inflammatory response.

## Introduction

Ulcerative colitis (UC), a chronic nonspecific intestinal inflammatory disease, clinically manifests as abdominal pain, bloody diarrhoea and various degrees of systemic symptoms. In recent decades, its incidence and prevalence have been continuously rising in Asia, and it predominantly affects young adults^1^. Most people believe that the occurrence and development of UC is caused by combining the effects of the intestinal barrier, gut microbiota, and mucosal immunity^2^. Studies have proven that the gut microbiota in UC patients remains dysbiosis. Disordered gut microbiota as a pathogenic factor leads to an impaired intestinal barrier and promotes the onset of UC, and targeting gut microbiota can also be used as a means to treat UC.

Herbal medicine has been used for thousands of years and has a good therapeutic effect in many diseases by achieving multiple goals in clinical treatment. Although the effectiveness of herbal medicine has been confirmed, the mechanism of its action on the body is still unclear.

As a traditional Chinese medicine, Abelmoschus Manihot is often used as the main drug in the treatment of chronic kidney disease (CKD) and other inflammatory diseases, which has remarkable antiinflammation, analgesia and antioxidant activities^3^. In our previous study, the total flavone of Abelmoschus Manihot was analysed by high performance liquid chromatography (HPLC). The results showed that TFA comprises eight flavone glycosides, including quercetin‑3‑O‑robinobioside, gossypetin‑3‑O‑glucoside, quercetin‑3′‑O‑glucoside, isoquercetin, hyperoside, myricetin, gossypetin and quercetin^4^. Animal experiments have verified that TFA has a therapeutic effect on UC^4,5^. However, its effect on intestinal flora and intestinal barrier function has not been proven. Therefore, we hypothesized that TFA, as a prebiotic, alleviates dextran sulfate sodium (DSS)-induced colitis by regulating the intestinal flora.

Our results suggested that TFA protected intestinal barrier integrity, inhibited the inflammatory response and significantly improved DSS-induced colitis by regulating intestinal flora and increasing the abundance of *Akkermansia muciniphila* (*A. muciniphila*). Its therapeutic effect was similar to that of PAT, and a high dose was better than a low dose. To the best of our knowledge, this is the first study on the effect of TFA on the intestinal flora in colitis and the promotion of *A. muciniphila *in vitro. It is expected that this work will provide experimental evidence for the application of TFA in UC. In addition, it may provide support for the development of potential prebiotics.

## Materials and methods

### Drugs and reagents

TFA was extracted from flowers of *Abelmoschus manihot* by the Jiangsu Provincial Hospital of Traditional Chinese Medicine, Nanjing, China. *Abelmoschus Manihot* flowers were purchased from Anhui Xiehecheng Co., Ltd. (Batch No 20092701). The source and production process of the *Abelmoschus Manihot* flowers were in accordance with Chinese Pharmacopoeia standards (2015 version). Fengyu Zhu identified *Abelmoschus Manihot* flowers in the Department of Pharmacy, Jiangsu Provincial Hospital of Traditional Chinese Medicine. *Abelmoschus Manihot* flowers were immersed in 75% ethanol for 60 min, refluxed for 60 min at 90 °C, and then filtered with analytical filter paper. Finally, rotary evaporation was used to evaporate the extracts under vacuum at 60 °C^6^. DSS (molecular weight of 36–50 kDa) was provided by German MP Biopharmaceutical Company. Prednisone acetate tablets (PAT) were purchased from Cisen Pharmaceutical Co. Ltd. (Jining, Shandong, China). Mucin (from porcine stomach) was purchased from Sigma (USA). TRIzol reagent was purchased from Life Technologies Inc., Grand Island, NY, USA.

### Animals

Six-week-old male C57BL/6J mice were purchased from Beijing Si Pei Fu Laboratory Animal Technology Co. Ltd. The mice were raised in the laboratory of Basic Pharmacology, Affiliated Hospital of Nanjing University of Chinese Medicine (Nanjing, Jiangsu, China). Sterilized standard rodent chow food and sterilized water were not restricted during the experiment, the temperature was controlled at 23 ± 1 °C, the humidity was controlled at 50 ± 5%, and the light system was set at 12 h/day. The care and use of the animals followed the animal welfare guidelines, and all the experimental protocols were approved by the Institutional Animal Care and Use Committee of Nanjing University of Chinese Medicine.

### Induction of colitis and treatment

**Figure 1:**
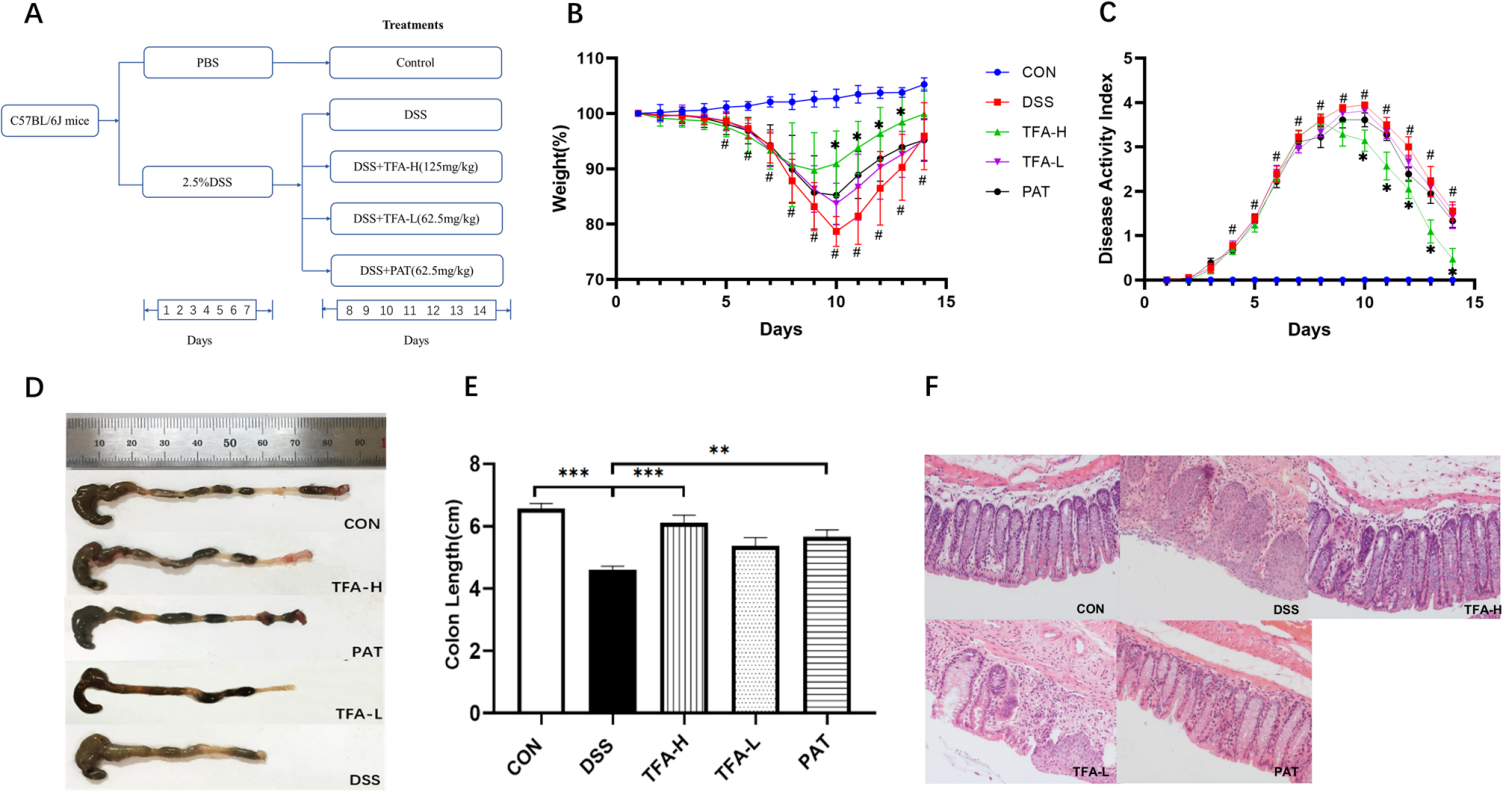
TFA ameliorated the symptoms of DSS-induced colitis in mice. (**A**) Schematic diagram of the experimental design. (**B**) Weight changes from day 1 to 14. (**C**) DAI score from day 1 to 14. *P < 0.05, compared with the DSS group; #* P* < 0.05, CON versus DSS group. (**D**) Macroscopic appearances of colon tissues. (**E**) The lengths of colon. Data are presented as the mean ± S.E.M. n = 6–8/group *P < 0.05, **P < 0.01, ***P < 0.001. (**F**) Histological changes (H&E staining images of colonic sections at original magnification 200×).

As shown in Fig. 1A, colitis was induced by 2.5% DSS in the drinking water ad libitum for 7 consecutive days (days 1–7). The mice were randomly allocated after modelling (n = 6–8). The mice were supplemented daily with 200 µL of phosphate buffered saline (vehicle), TFA (125 mg/kg, 62.5 mg/kg) or PAT (2.5 mg/kg) by intragastric gavage for 7 consecutive days (days 8–14). Mice were sacrificed by cervical dislocation on the 15th day, and the colon was obtained to measure the colon length. One centimetre of distal colon tissue was collected for histologic examination. The remaining intestinal tube was cut longitudinally, the intestinal mucosa was quickly scraped with a glass slide, and the tube was stored at − 80 °C for RNA extraction. The serum samples were collected on the 15th day. Before the mice were sacrificed on the 15th day, blood was collected through the retro-orbital blood after fully anesthesia with amobarbital. Blood was left to set at room temperature for 30 min and then centrifuged at 3500 rpm for 10 min at 4 °C to obtain serum.

### Disease activity index (DAI)

The changes in DAI were measured using the following criteria: (1) weight loss (%), (2) stool consistency and (3) blood in faeces as previously described (Table 1)^7^.

**Table 1:** Disease activity index (DAI).

Weight loss (%)	Stool consistency	Occult blood	Score
None	Normal	Negative	0
1–5	–	–	1
5–10	Loose stools	Haemoccult +	2
10–20	–	–	3
> 20	Diarrhoea	Gross bleeding	4

### Haematoxylin and eosin (H&E) Staining

Distal colon specimens were fixed for 48 h in 4% formalin after mice were sacrificed. Then, the distal colon specimens were paraffin-embedded. Finally, the sections were segmented and stained with haematoxylin and eosin, and pathological changes were observed with a light microscope.

### Immunohistochemical staining

First, paraffin sections were dewaxed in water; antigen repair was performed, and endogenous peroxidase was blocked. The sections were blocked in serum, which was followed by primary antibody application, secondary antibody application, DAB colour development, nuclear staining, dehydration and sealing. Finally, the positive expression of mucin-2 (MUC2), Kruppel-like factor 4 (KLF4) and zonula occludens-1 (ZO-1) in colonic mucosal epithelial cells was observed under a microscope.

### Assessment of cytokine level in serum

The levels of tumour necrosis factor-α (TNF-α), interleukin-1β (IL-1β) and interleukin-6 (IL-6) were measured using commercial ELISA kits (Jin Yi Bai Biological Technology Co. Ltd., Nanjing, China) according to the manufacturer’s instructions.

### Quantitative real-time polymerase chain reaction (qPCR)

Total RNA was extracted from colon tissues using TRIzol reagent, and the concentration of RNA was measured and then reverse transcribed according to the manufacturer's instructions using a HiScript 1st Strand cDNA Synthesis Kit (Vazyme, Nanjing, China). cDNA was used for qPCR using SYBR Green Master Mix (Service, Wuhan, China) on an ABI 7500 Fast Real-Time PCR System (Applied Biosystems). Relative amounts of mRNA were calculated using the 2^−ΔΔCT^ method, and GAPDH served as the housekeeping gene. The primer sequences are shown in Table 2. The abundance of *A. muciniphila* in stool samples was quantified by quantitative PCR. All procedures were performed according to Everard et al.^8^.

**Table 2:** Primers used in the real-time PCR assays.

Gene	Primer sequences (5′–3′)
**TNF-α**	
Forward	CACCACGCTCTTCTGTCTACTG
Reverse	GGGCTACAGGCTTGTCACTC
**IL-1β**	
Forward	CTCGTGCTGTCGGACCCAT
Reverse	GCTTGTGCTCTGCTTGTGA
**IL-6**	
Forward	GAGGATACCACTCCCAACAGACC
Reverse	AAGTGCATCATCGTTGTTCAT
**IL18**	
Forward	GTGAACCCCAGACCAGACTG
Reverse	CCTGGAACACGTTTCTGAAAGA
**IL-17a**	
Forward	GTTAGGGTGCTTTAGGTCC
Reverse	TAACAATGAGTTTCTGTACG
**CCL2**	
Forward	TGCCCTAAGGTCTTCAGCAC
Reverse	AAGGCATCACAGTCCGAGTC
**MUC2**	
Forward	TGCCCACCTCCTCAAAGAC
Reverse	TAGTTTCCGTTGGAACAGTGAA
**KLF4**	
Forward	CAGGATTCCATCCCCATCCG
Reverse	GAGAGGGGACTTGTGACTGC
**ZO-1**	
Forward	GGGGCCTACACTGATCAAGA
Reverse	TGGAGATGAGGCTTCTGCT
**GAPDH**	
Forward	AGAACATCATCCCTGCATCC
Reverse	CTGGGATGGAAATTGTGAGG

### 16S rDNA gene high-throughput sequencing

The V3-V4 variable region of the bacterial 16S rRNA gene was amplified by F338 (5′-ACTCCTACGGGAGGCAGCA-3′) and R806 (5′-GGACTACHVGGG TWTCTAAT-3′). On the Illumina MiSeq platform, the extracted PCR products were analysed by isomolecular 250-bp double-terminal sequencing. The original pyrophosphate sequence was uploaded to the NCBI Data Center database SRA (Sequence Read Archive). High-quality sequence merge overlaps generated fastq files. QIIME (version 1.9.1, https://qiime.org/) software was used to multichannel decode and quality control filter the fastq file output. All sequencing and bioinformatics analysis were performed using the Omicsmart online platform (http://www.omicsmart.com).

### Bacterial strains and growth curve

*A. muciniphila* strain ATCC was cultured in brain heart infusion (BHI) medium in tubes at 37 °C in an anaerobic chamber. *A. muciniphila* were collected in log phase and diluted with sterile phosphate-buffered saline (PBS) to 3 × 10^8^ colony-forming units/mouse for gavage. *A. muciniphila* freshly prepared every day for gavage. To acquire the growth curve of *A. muciniphila*, different concentrations of TFA were added to BHI medium, and then an *A. muciniphila* suspension was added to a final concentration of bacteria 10^6^ cfu /ml. The growth profile was evaluated by intermittently measuring absorbance at 600 nm every 5 hours^9^. Mucin was added to the final concentration of 4 g/L. TFA was dissolved in PBS. Each experiment was repeated three times.

### Statistical analysis

Graphing was performed using GraphPad Prism (version 9.0, https://www.graphpad.com). One-way ANOVA analysis of variance was applied to compare differences between multiple groups. When only two groups were compared, Student’s t-test was conducted. Non-parametric, were tested by the Mann–Whitney test. A value of *P* < 0.05 indicated that the difference was statistically significant. All plots are shown as the mean ± standard error of the mean (S.E.M). *P* < 0.05 was considered statistically significant.

### Statement

We confirm that all methods in our study are reported in accordance with ARRIVE guidelines.

## Result

### Effects of TFA on damage in the colon of DSS-induced colitis mice

As shown in Fig. 1, the DSS group showed significant weight loss, diarrhoea, haematochezia and other colitis symptoms (Fig. 1B,C). Treatment with TFA (62.5 and 125 mg/kg) significantly improved weight loss and decreased the DAI score in a dose-dependent manner. As shown in Fig. 1D,E, compared to that of the control group, the colon length was markedly shortened in the DSS group (*P* < 0.01). In contrast, TFA-H (*P* < 0.01) and PAT (*P* < 0.01) were used to significantly improve the colonic shortening induced by DSS. As shown in Fig. 1F, in the control group, the colon tissue structure was intact. In contrast, the DSS group was characterized by inflammatory cell infiltration, epithelial cell destruction and mucosal thickening. Consistent with the symptom observations, compared to the DSS group, the TFA-H and PAT groups had significantly restored intestinal epithelial structure and reduced severe inflammation. These results indicated that TFA-H has an obvious protective effect on DSS-induced colitis, which was similar to that of PAT and superior to that of TFA-L.

### Effects of TFA on the production of inflammatory cytokines

**Figure 2:**
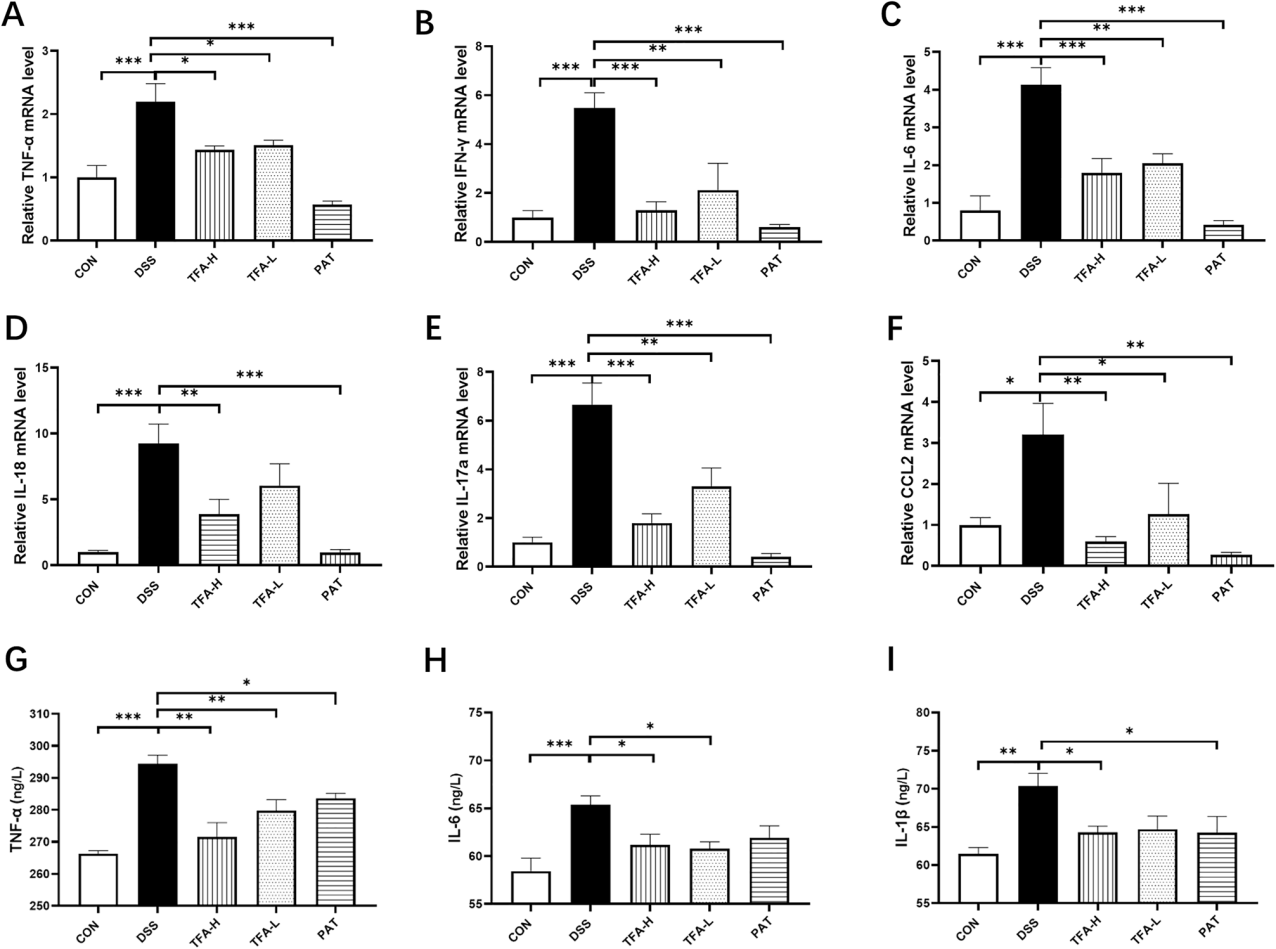
Inflammatory cytokines in the mouse colon after treatment with DSS and administration of TFA and PAT. (**A**–**F**) mRNA quantification of proinflammatory cytokines (TNF-α, IL-6, IFN-γ, IL-18 and IL-17a) and chemokines CCL2 using real-time RT–PCR. (**G**–**I**) Determination of inflammatory cytokine protein production using ELISA. The serum samples were collected on the 15th day. The data present the mean ± S.E.M. and n = 5/group. *P < 0.05, **P < 0.01, ***P < 0.001, #*P* < 0.0001.

Inflammatory molecules are involved in the processes that occur in colitis. To elucidate the inflammatory response in DSS-induced mice, various proinflammatory cytokines were measured in colon tissues at the mRNA level. As illustrated in Fig. 2A–F, the levels of proinflammatory cytokines, including TNF-α, IL-6, interferon-γ (IFN-γ), interleukin-18 (IL-18) and interleukin-17a (IL-17a), were significantly increased in DSS-induced colitis mice (*P* < 0.05 vs. CON). These elevated proinflammatory cytokines were all decreased by TFA in a dose-dependent manner. Chemokine ligand 2 (CCL2) was significantly increased by DSS treatment compared with that in the control group. Moreover, TFA and PAT significantly decreased CCL2 compared with the DSS group (Fig. 2F). Proinflammatory cytokines were also measured in serum at the protein level. Quantification of specific cytokines using ELISA showed the same patterns in the regulation of the production and secretion of proinflammatory cytokines (Fig. 2G–I). Notably, TFA-H (125 mg/kg) exhibited a pronounced effect in suppressing these inflammatory cytokines.

### TFA improved intestinal barrier integrity in DSS‑induced colitis mice

**Figure 3:**
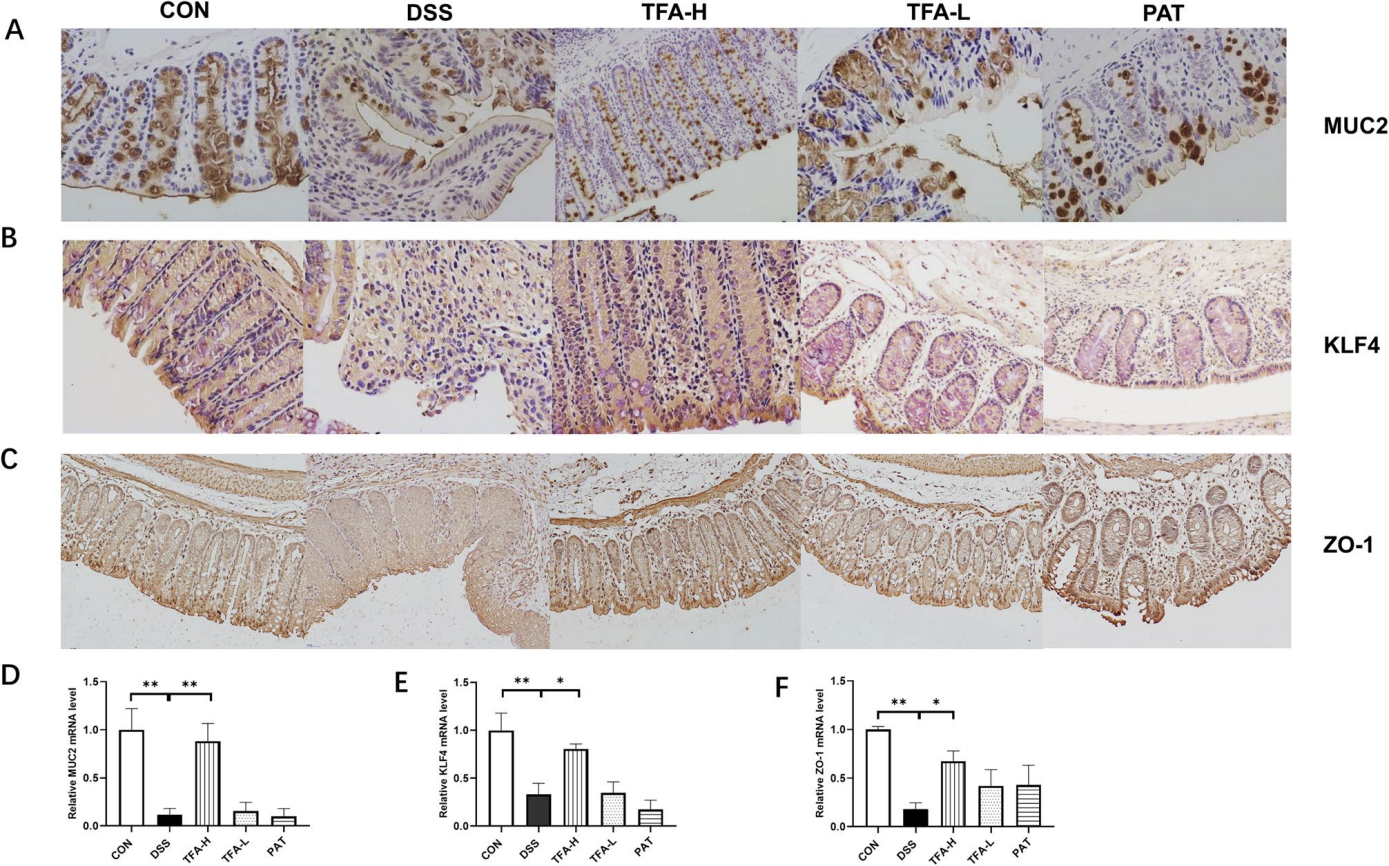
Recovery of mucus- and tight junction-associated proteins from the colons of DSS-induced colitis mice by TFA and PAT. (**A**) The number of colonic MUC2-positive cells in DSS-treated mice. Representative images (× 200 magnification, scale bar 50 μm). (**B**) The number of colonic KLF4-positive cells in DSS-treated mice. Representative images (× 200 magnification, scale bar 50 μm). (**C**) The number of colonic ZO-1-positive cells in DSS-treated mice. Representative images (× 200 magnification, scale bar 50 μm). (**D**) mRNA quantification of MUC2. (**E**) mRNA quantification of KLF4. (**F**) mRNA quantification of ZO-1. The data present the mean ± S.E.M. and n = 5/group. *P < 0.05, **P < 0.01, ***P < 0.001, #*P* < 0.0001.

To understand the effect of TFA on the intestinal barrier integrity of the mice with DSS‑induced colitis, the expression of MUC2, KLF4 and ZO-1 in the colon was determined by qPCR and immunohistochemical staining. Immunohistochemical staining results showed that the content of MUC2-, KLF4- and ZO-1-positive cells in DSS‑induced mice was significantly lower than that in the control group (Fig. 3A–C). TFA treatment significantly increased MUC2-, KLF4- and ZO-1-positive cells. MUC2, KLF4 and ZO-1 mRNA expression was decreased in the colon tissue of the DSS group. In contrast, TFA-H treatment returned the expression of MUC2, KLF4 and ZO-1 to normal levels (Fig. 3D–F). This result suggested that TFA treatment protects the epithelial barrier by recovery or even enhancement of mucus and tight junction-associated proteins in DSS-induced colitis.

### TFA modulated the structure of gut microbiota

**Figure 4:**
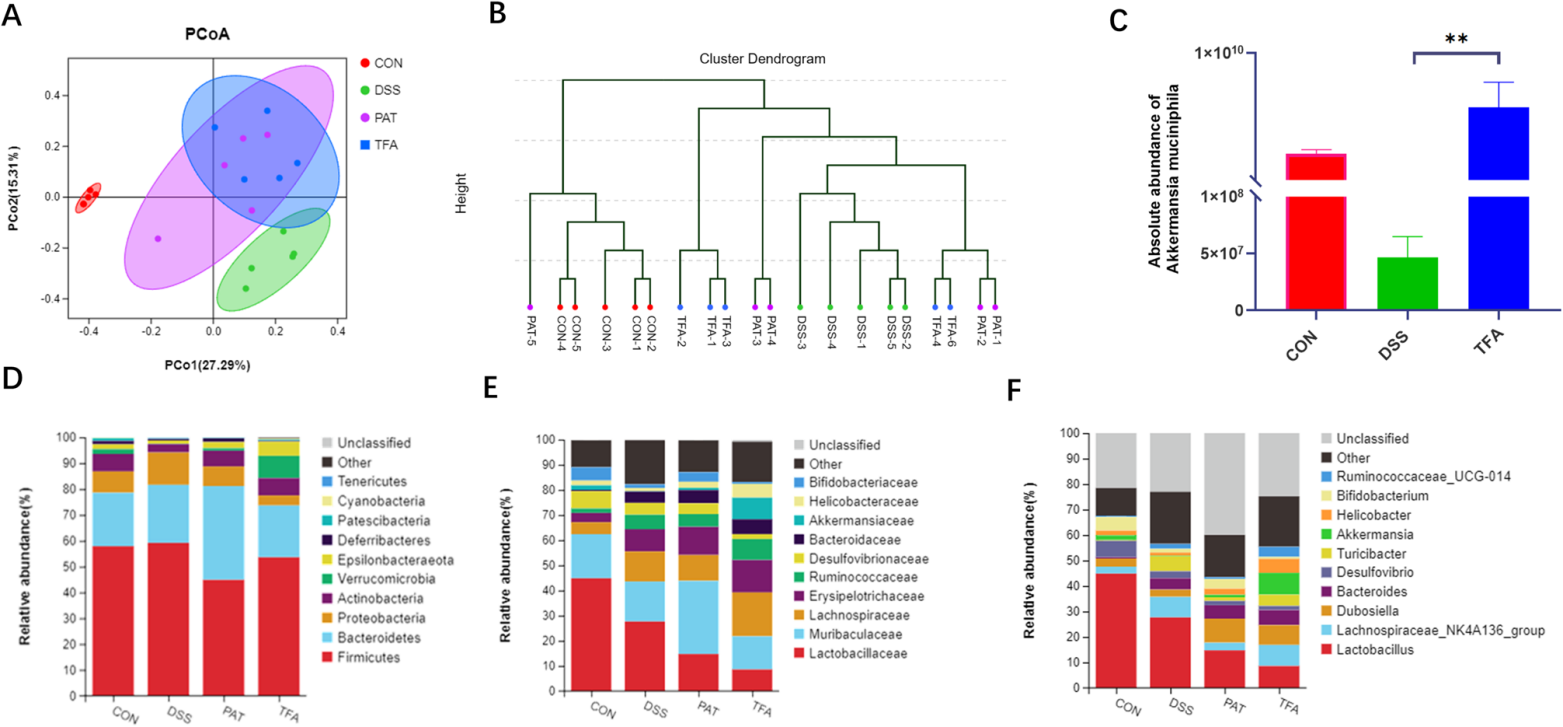
The composition of the gut microbiota in the treatment of DSS and administration of TFA-H and PAT. (**A**) PCoA plot of OTU data. (**B**) Cluster analysis based on Bray–Curtis metric distances. (**C**) Absolute abundance of *Akkermansia muciniphila* in faecal content by qPCR. Data are shown as means ± S.E.M., **P < 0.01, n = 5/group. (**D**–**F**) Relative abundance of taxa at the phylum (**D**), family (**E**) and genus (**F**) levels.

To demonstrate whether TFA-H regulated DSS-induced gut microbial dysbiosis, high-throughput sequence analysis of the bacterial 16S rRNA V3-V4 region was conducted on stool samples. Principal coordinates analysis (PCoA) plots were calculated from Bray–Curtis metric distances to evaluate the composition of the community, and the results revealed a clear separation between each group (Fig. 4A). The system clustering tree indicated marked differences among the four groups. The TFA-H group and DSS group clustered separately, demonstrating that treatment with TFA-H inhibited DSS-induced gut microbiota dysbiosis in mice (Fig. 4B).

The most abundant taxa at the phylum, family and genus levels are shown in Fig. 4D–F. Compared with the control group, the abundance of *Verrucomicrobia* was lower in the DSS group, while the abundances of *Tenericutes* and *Proteobacteria* were higher, which was consistent with a previous report^10,11^. At the genus level, the abundances of *Clostridium*, *Parabacteroides*, *Ruminococcus* and *Romboutsia* were remarkably increased, whereas those of *A. muciniphila* and *Lactococcus* were significantly decreased in the DSS group compared to the control group (*P* < 0.05). Following treatment with TFA, the abundance of *Tenericutes* and *Proteobacteria* nearly returned to normal levels. TFA treatment significantly increased the relative abundance of *A. muciniphila*, which belongs to *Verrucomicrobia*. PCR also proved the increase in absolute abundance of *A. muciniphila* in the TFA group (Fig. 4C). Spearman correlation analysis showed that the abundance of *A. muciniphila* is negatively correlated with IL-6, IFN-γ and CCL2, and positively correlated with MUC2, ZO-1 and KLF4 (Supplementary Fig. S2A). However, the low-dose TFA (62.5 mg/kg) did not increase the abundance of *A.muciniphila* (Supplementary Fig. S2B,C). The microbial flora structure was favourably harmonized by treatment with TFA-H.

**Figure 5:**
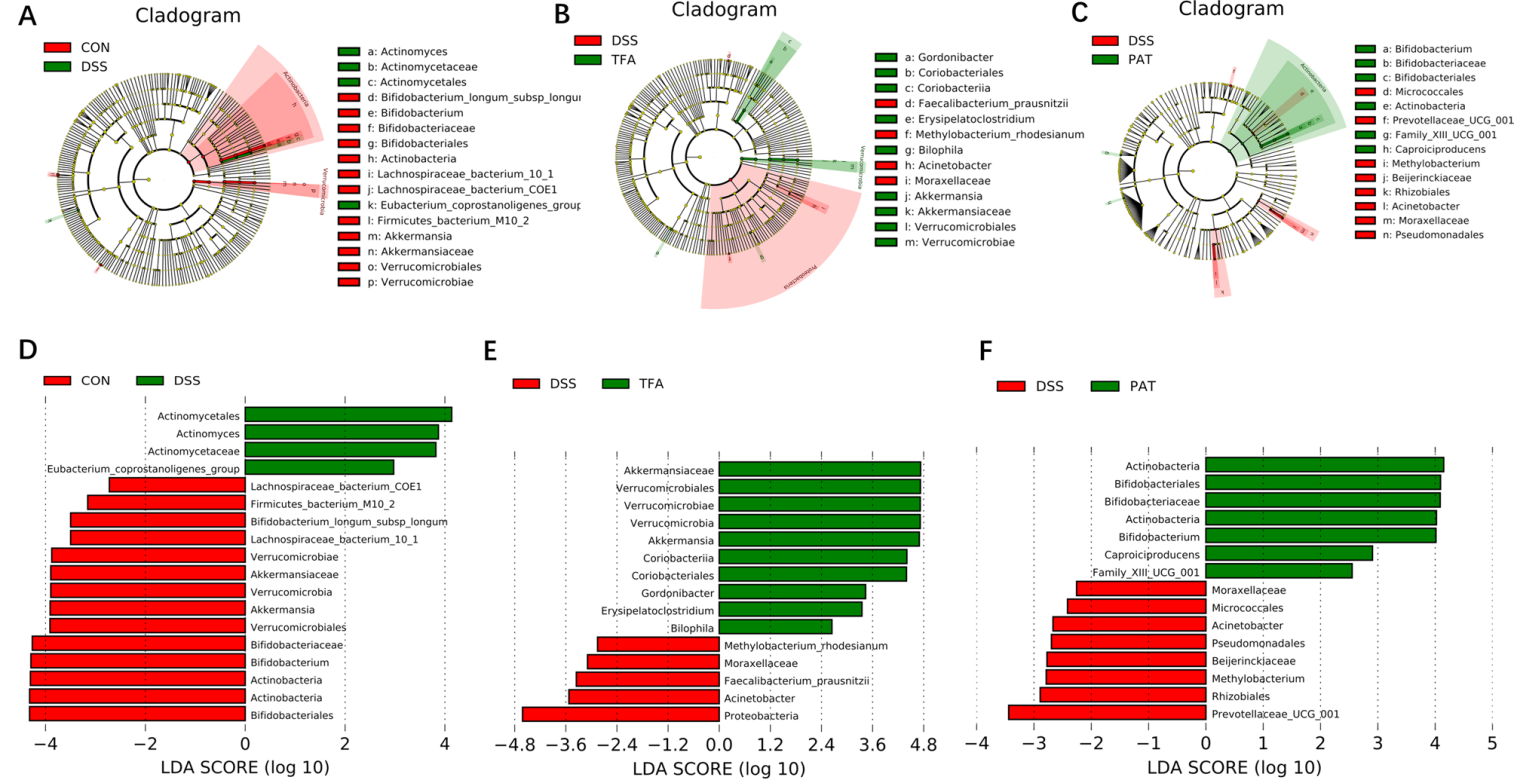
Characteristics of microbial community composition using LEfSe analysis. (**A**) LEfSe cladogram represents the taxa enriched in the DSS group (green) and control group (red). (**B**) Discriminative biomarkers with an LDA score > 2 between the DSS group (green) and control group (red). (**C**) LEfSe cladogram represents taxa enriched in the DSS group and TFA group. (**D**) Discriminative biomarkers with an LDA score > 2 between the DSS group (red) and TFA group (green). (**E**) LEfSe cladogram represents taxa enriched in the DSS group and PAT group. (**F**) Discriminative biomarkers with an LDA score > 2 between the DSS group (red) and PAT group (green), n = 5/group.

Furthermore, LefSe (LDA effect size) analysis was used to identify dominant flora in each group (Fig. 5). Compared with the control group, the gut microbiota *Actinomycetales* (LDA = 3.87) and *Ruminococcaceae* (LDA = 2.973) were enriched, and there was a depletion of *Bifidobacterium* (LDA = 4.29) and *A. muciniphila* (LDA = 3.90) in the DSS group. The TFA-H group showed significant selective enrichment of *A. muciniphila* (LDA = 4.69), *Gordonibacter* (LDA = 3.43) and *Erysipelatoclostridium* (LDA = 3.35). PAT demonstrated a significant effect on *Bifidobacterium* (LDA = 4.01), *Family_XIII_UCG_001* (LDA = 2.55), and *Ruminococcaceae* (LDA = 2.90). The results indicated that TFA-H alleviated the disorder of the gut microbiota in DSS-induced mice, especially increasing the abundance of *A. muciniphila*.

### TFA promoted *A. muciniphila* growth in vitro

**Figure 6:**
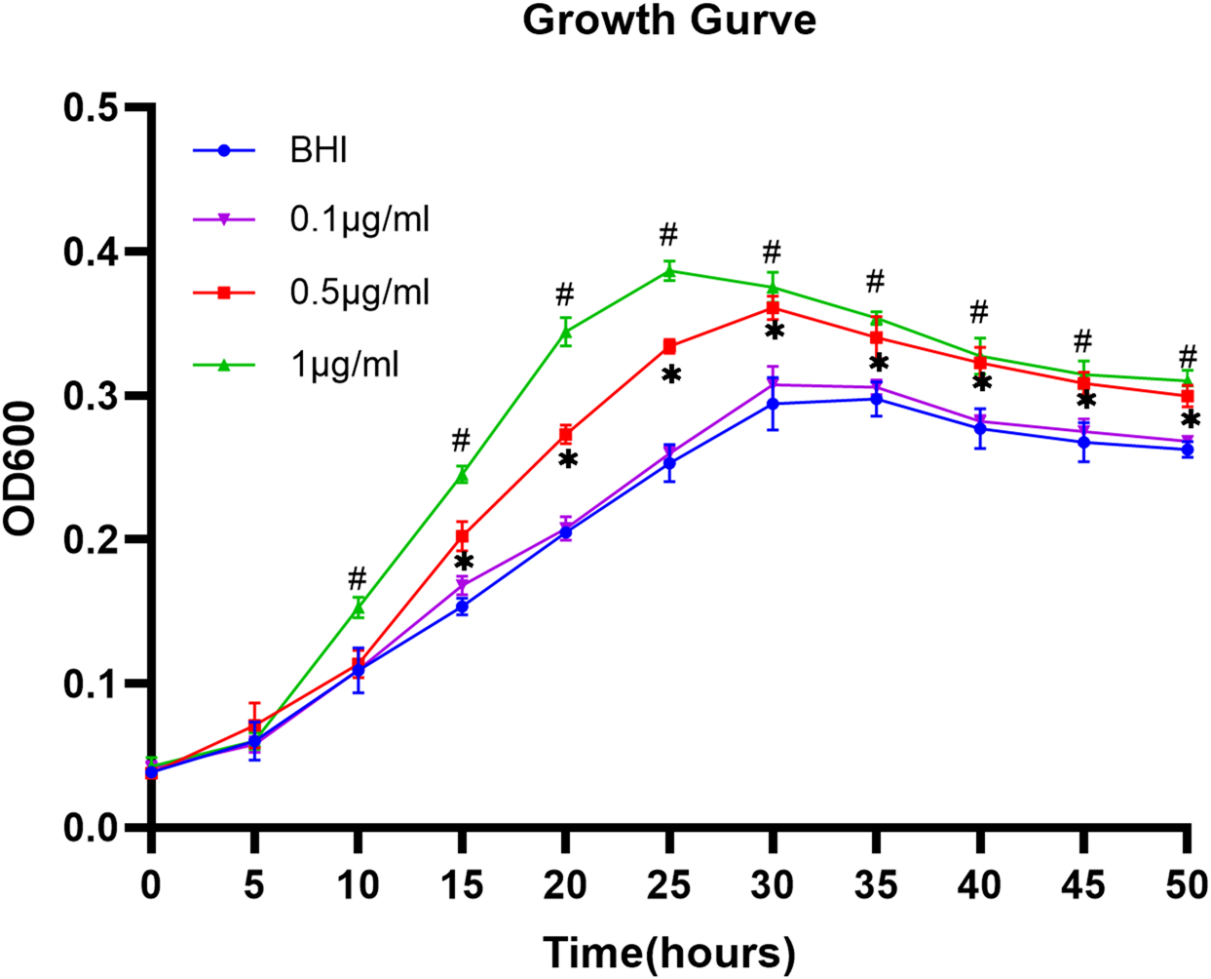
Growth curves of *Akkermansia muciniphila* under different conditions. Data are presented as the mean ± S.E.M. *P < 0.05, BHI compared with TFA (0.5 µg/mL), #*P* < 0.05, BHI versus TFA (1 µg/mL).

To examine whether TFA directly promoted the growth of *A. muciniphila *in vitro, the growth curve of *A. muciniphila* was monitored in BHI medium with mucin (Fig. 6). TFA (1 µg/mL and 0.5 µg/mL) directly promoted the growth of *A. muciniphila* in vitro. However, the much lower(0.1 μg/ml) concentrations cannot promote the growth of *A. muciniphila*. When TFA (1 µg/mL or 0.5 µg/mL) was added, *A. muciniphila* grew faster at log phase and plateaued at a much higher cell density. We added much higher concentration of TFA (10 µg/mL and 100 µg/mL) and the results showed that the promotion effect of TFA (10 µg/mL) is inferior to that of TFA(1 µg/mL). TFA (100 µg/mL) inhibited the growth of *A. muciniphila* (Supplementary Fig. S3). Thus, we inferred that TFA promoted the growth of *A. muciniphila* in a dose-dependent manner within a certain concentration range.

### A. muciniphila alleviated colitis in mice

**Figure 7:**
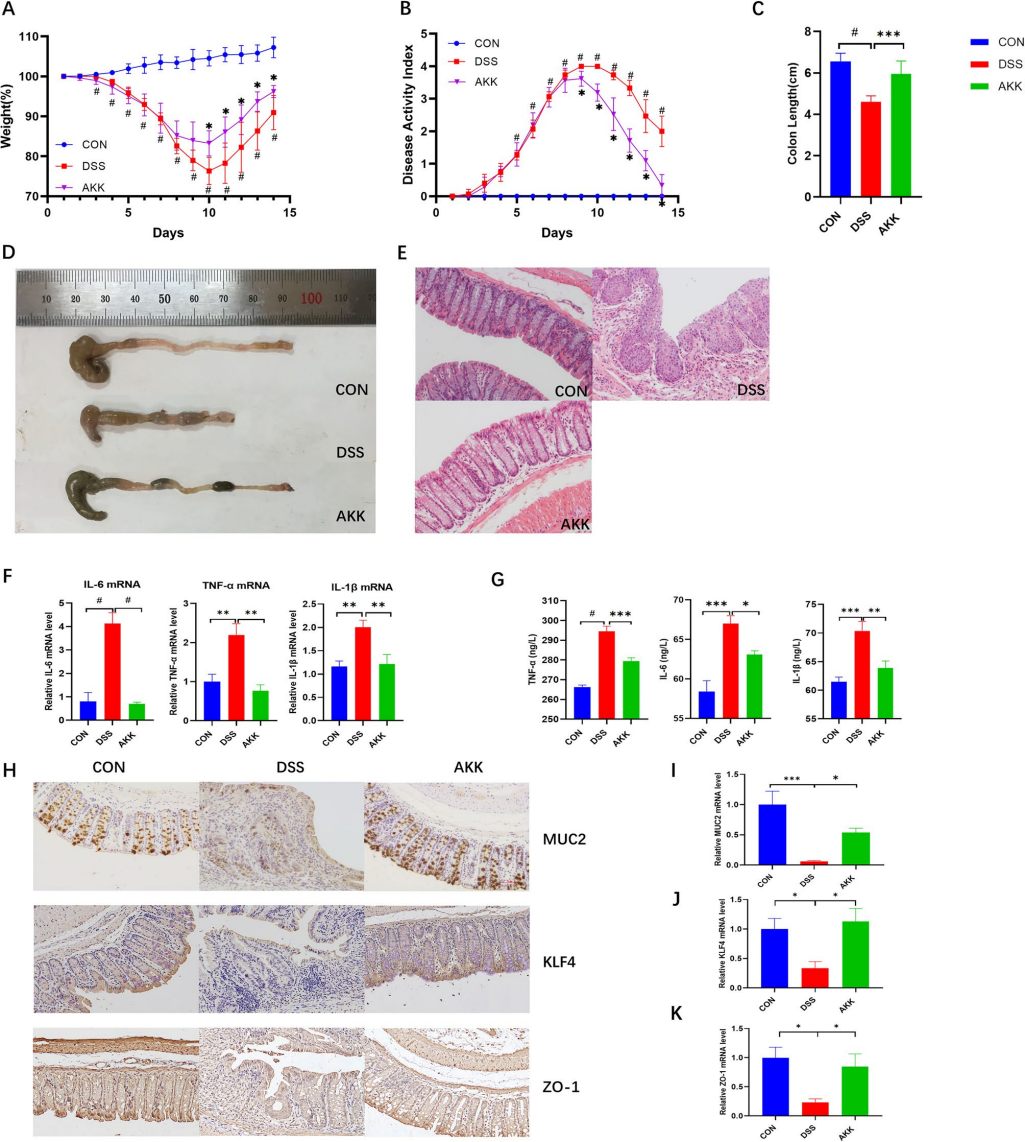
AKK ameliorated the symptoms of DSS-induced colitis in mice. (**A**) Daily bodyweight changes from day 1 to 14. (**B**) DAI score from day 1 to 14. *P < 0.05, compared with the DSS group; #*P* < 0.05, CON versus DSS group. (**C**) The lengths of colon. Data are presented as the mean ± S.E.M. and n = 6–8/group. *P < 0.05, **P < 0.01, ***P < 0.001, #*P* < 0.0001. (**D**) Macroscopic appearances of colon tissues. (**E**) Histological changes. (**F**) mRNA quantification of proinflammatory cytokines (TNF-α, IL-6, IL-1β) using real-time RT–PCR. (**G**) Determination of protein production of inflammatory cytokines using ELISA. The data present the mean ± S.E.M. Asterisks denote significance versus DSS group by one-way ANOVA (*P < 0.05, **P < 0.01, ***P < 0.001). (**H**) The number of colonic MUC2-, KLF4-, and ZO-1-positive cells in DSS-treated mice. Representative images (× 200 magnification, scale bar 50 μm). (**I**–**K**) mRNA quantification of MUC2, KLF4 and ZO-1.

To confirm whether *A. muciniphila* played an essential role in DSS-induced colitis. We treated DSS-induced mice daily with *A.* muciniphila (3×10^8^) or PBS for 1 week after DSS modelling. Treatment with *A. muciniphila* relieved DSS-induced colitis, which was evidenced by reduced weight loss, colon length shortening and histological damage (Fig. 7A–E). Serum and colon tissue levels of inflammatory cytokines (TNF-α, IL1β, IL6) decreased as a result of *A. muciniphila* treatment (Fig. 7F–G). *A. muciniphila* treatment increased the expression of the tight junction proteins ZO-1, MUC2 and KLF4, supporting a potential role in the regulation of intestinal barrier integrity (Fig. 7H–K). In summary, *A. muciniphila* ameliorated DSS-induced colitis, improved macroscopic and histological damage, decreased inflammatory cytokines, and protected intestinal barrier integrity.

## Discussion

Studies have shown that the gut microbiota of UC patients is out of balance^12^. Metagenomic research has shown that the abundance and diversity of the gut microbiota of UC patients are reduced^2^. Recent studies have shown that certain dietary agents, spices, oils, and dietary phytochemicals that are consumed regularly possess beneficial effects in regulating gut microbiota^13,14^. The aim of this study was to characterize the effects of TFA on DSS-induced colitis, mainly focusing on the composition of the gut microbiota and intestinal barrier in mice with DSS-induced colitis.

In our study, TFA relieved the symptoms of weight loss and colon length shortening in DSS-induced mice. Compared with PAT, TFA-H has advantages in weight and DAI score. In terms of colonic histopathology, DSS-induced colitis in mice exhibited serious injuries, with the loss of histological structure, disruption of the epithelial barrier, a pronounced decrease in the number of crypts, and marked infiltration of granulocytes and mononuclear cells into the mucosa and submucosa. Compared to the DSS group, the TFA group was observed to effectively reduce histologic inflammation. Notably, TFA-H exhibited a similar effect to PAT and exerted a superior effect to TFA-L.

Animal experiments and clinical studies have found that an increase in proinflammatory cytokines further damages intestinal mucosal barrier function through activation of the NF-κB signalling pathway. Our previous study showed that TFA could markedly inhibit the release of intestinal inflammatory cytokines in Crohn's disease rats induced by 2,4,6-trinitrobenzene sulfonic acid, improve intestinal inflammation and ameliorate colitis symptoms^6^. In this study, we demonstrated that supplementation with TFA significantly decreased the mRNA expression of inflammatory cytokines (TNF-α, IL-6, IL-18, IL-17a, IFN-γ) and chemokines (CCL2) in colon tissue. TFA also decreased the protein expression of inflammatory factors (TNF-α, IL-6, IL-1β) in the serum. Notably, TFA-H exerted superior anti-inflammatory effects to TFA-L.

The intestinal mucosal barrier plays an important role in the pathogenesis of colitis^15^. The function of the intestinal mucosal barrier mainly refers to the isolation of the intestinal lumen from the environment to prevent the invasion of bacteria and toxic substances. The intestinal mucus layer is a protective gel-like substance covering the surface of the intestinal mucosa, which is the first barrier in the intestinal lumen. A large amount of mucin synthesized and secreted by goblet cells is the most important substance that constitutes the mucus layer of the intestine^16^. The destruction of the mucus layer and the pathological changes of goblet cells are closely related to the progression of colitis^17^. Our experiment showed that DSS-induced colitis decreased the mRNA expression of KLF4, MUC2 and ZO-1 in colon tissue. KLF4 is a zinc finger transcription factor expressed in differentiated epithelial cells of the intestine and is widely involved in the regulation of cell proliferation, differentiation and embryonic development, especially in the proliferation of goblet cells^18^. Studies have shown that goblet cells are not fully developed and that the expression of mucin MUC2 is abnormal in the colon tissue of KLF4^−/−^ mice^19^. The number of goblet cells in the colon tissue of intestine-specific KLF4 deletion mice was significantly reduced^20^. In the mucus layer secreted by goblet cells, the highest content of intestinal epithelial cell proliferation and differentiation is mucin MUC2. MUC2 effectively blocks pathogens in the intestinal lumen from invading intestinal epithelial cells. MUC2 also provides a habitat and nutrients for symbiotic bacteria in the intestine. The mucus layer disappeared in the colon of MUC2^−/−^ mice, direct contact between the gut microbiota and intestinal epithelial cells triggered an inflammatory response, and spontaneous colitis eventually formed^21,22^. ZO-1 is crucial for connecting individual epithelial cells and maintaining the integrity of the epithelium. The reduction of ZO-1 interrupts the assembly of tight junctions by inhibiting the recruitment of other components^23^. Destruction of intestinal barrier integrity causes pathogens in the intestinal lumen to invade intestinal epithelial cells, increases the immune response of intestinal epithelial cells, and ultimately strengthens the intestinal inflammatory response. Treatment with TFA improved intestinal mucosal barrier integrity by promoting the production of MUC2, KLF4 and ZO-1.

A previous study showed that the composition of the intestinal flora is altered in UC patients or in DSS-treated mice^24^. In addition, the bacteria associated with the mucosa increase the mucus layer thickness and promote the repair of the intestinal barrier^25^. Several studies have focused on the clinical improvement of DSS-induced colitis by using probiotics and antibiotics to modulate the commensal microbiota^26^. Dietary flavone could contribute to the maintenance of intestinal health by preserving the gut microbial balance through the stimulation of the growth of beneficial bacteria and the inhibition of pathogenic bacteria^27^. We found that the gut microbiota of mice with DSS was substantially changed during TFA-H or PAT treatment. In our study, a lower abundance of *A. muciniphila* and *Bifidobacterium* and a higher abundance of *Actinomyces, Escherichia coli* and *Proteobacteria* were observed in the DSS group than in the control group, which was consistent with previous reports. Studies have shown that the abundance of *Actinomycetes* in UC patients is significantly increased, and *Actinomyces* is also significantly increased in intramucosal carcinomas^28^. *Escherichia coli*, a conditional pathogenic bacterium, causes the disease to worsen by destroying intestinal barrier integrity^29^. Treatment with TFA-H reversed the intestinal dysbacteriosis caused by DSS. In addition, the abundance of *A. muciniphila* in TFA-H was significantly increased.

Our previous study found that in patients with UC, the abundance of *A. muciniphila* was significantly reduced. This is consistent with the results reported in the literature^30–32^. A prospective study unequivocally showed that 3 months of administration of *A. muciniphila* (10^10^ cfu/day) was safe and verified the feasibility and tolerance of *A. muciniphila* supplementation in humans. In our study, 1 week of treatment with *A. muciniphila* (3×10^8^ cfu/mouse/day) improved the colitis induced by DSS. Treatment with *A. muciniphila* significantly reduces the expression of inflammatory cytokines (TNF-α, IL-6, IL-1β) and protects the intestinal barrier by increasing the thickness of the mucus layer and the expression of tight junction proteins. Studies have shown that *A. muciniphila* is a protein-degrading anaerobic bacterium attached to the mucus layer of the intestine. It can promote the metabolism of the mucous layer, thereby creating a healthy environment for intestinal epithelial cells^8,33^. In vitro experimental studies have shown that *A. muciniphila* enhances the integrity of the intestinal epithelium and repairs the damaged intestinal mucosal barrier. This may be related to the metabolites of *A. muciniphila*. *A. muciniphila* maintains the homeostasis of the intestinal epithelium and inhibits the immune response of intestinal epithelial cells by degrading the host's intestinal mucus into short-chain fatty acids^34^. Previous study has shown that *A. muciniphila*, its outer membrane protein Amuc_1100 and the extracellular vesicles derived from *A. muciniphila* could protect the progression of DSS-induced colitis and maintain the integrity of the intestinal mucosal barrier. The mechanism by which *A. muciniphila* relieves colitis may be related to the interaction of Amuc_1100 with Toll-like receptor 2. Extracellular vesicles derived from *A. muciniphila* promote tight junction expression by activating AMPK^35,36^. A study certified that *A. muciniphila* promotes intestinal inflammation in a germ-free IL10^−/−^ mouse model of IBD^37^. However, a subsequent study showed that *A. muciniphila* was examined in gnotobiotic IL10^−/−^ mice and did not promote intestinal inflammation^38^. *A. muciniphila* plays a regulatory role in maintaining intestinal barrier integrity, host metabolism and other biological functions^39^.

In the in vivo experiment, PCR confirmed the increase (*P* < 0.01) in absolute abundance of *A. muciniphila* in mice treated with TFA-H (Fig. 4C). *In an *in vitro experiment, TFA promoted the growth of *A. muciniphila* in a dose-dependent manner within a certain concentration range.* A. muciniphila* grew faster at the logarithmic phase and at a higher cell density in the platform period. TFA may improve the intestinal barrier to relieve colitis through its prebiotic effect on *A. muciniphila.*

## Conclusion

The study showed that TFA treatment improved the colitis caused by DSS, at least partly through modulating gut microbiota and restoring the integrity of the intestinal barrier. Our study further suggested that TFA administration promotes the growth of *A. muciniphila*, which may be associated with this protective effect. We reveal that TFA, as a prebiotic of *A. muciniphila,* effectively treats colitis.

## Supplementary Information

Supplementary Information.

## Data Availability

Illumina amplicon sequences were submitted to the National Center for Biotechnology Information (NCBI) Sequence Read Archive (SRA) database under accession number PRJNA765530.
