# Supplementary material for: Total flavone of Abelmoschus Manihot improves colitis by promoting the growth of Akkermansia in mice
Source: Sci Rep. 2021 Oct 21;11:20787. doi: 10.1038/s41598-021-00070-7 (PMC8531128; doi:10.1038/s41598-021-00070-7)
Supplement: Supplementary file 1 — Supplementary Information. [file 41598_2021_70_MOESM1_ESM.docx]

Supplementary Figure S1


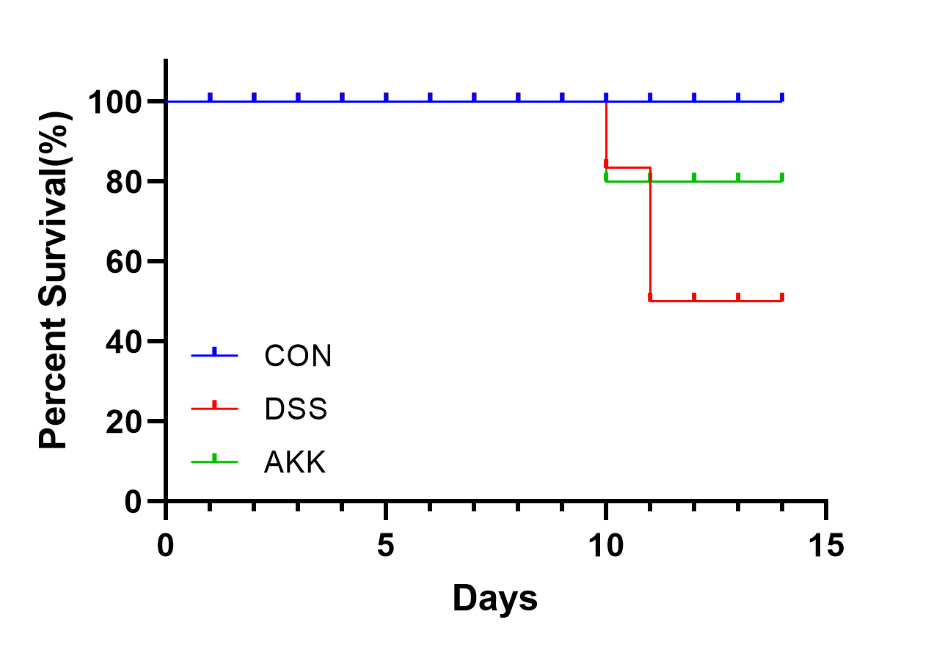

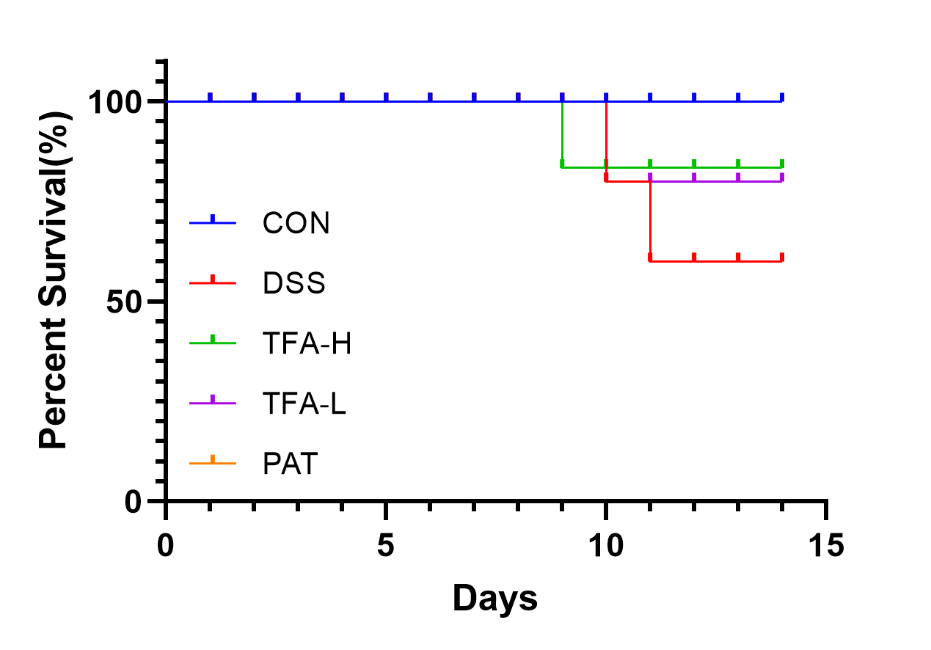
A B

Supplementary Figure1 The Survival curve of mice (A. Animal experiment 1, B.Animal experiment 2)

Supplementary Figure S2


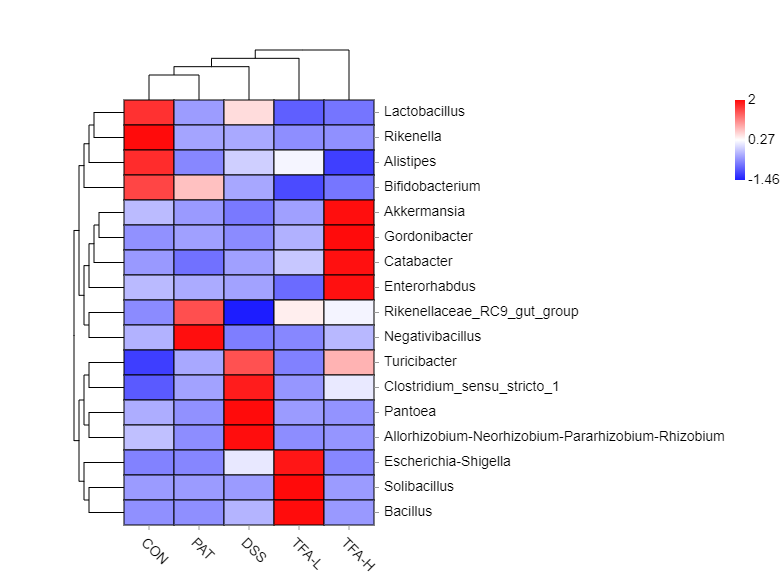

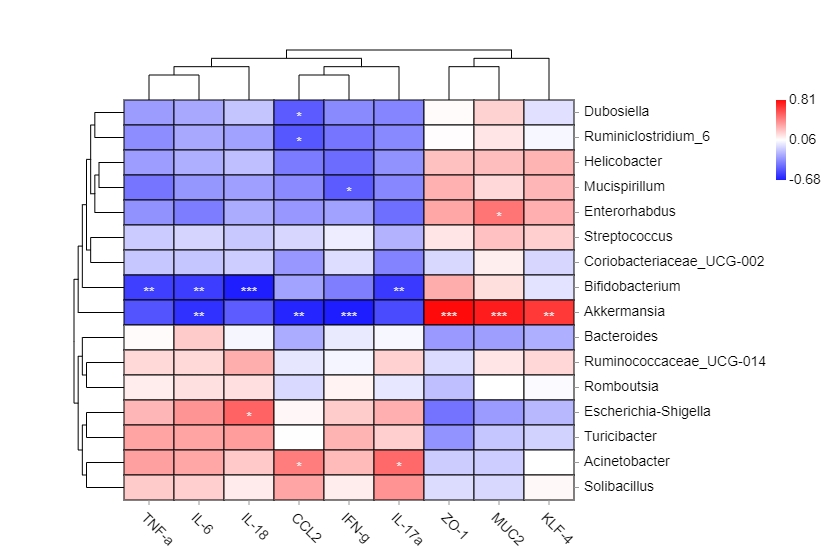
A B


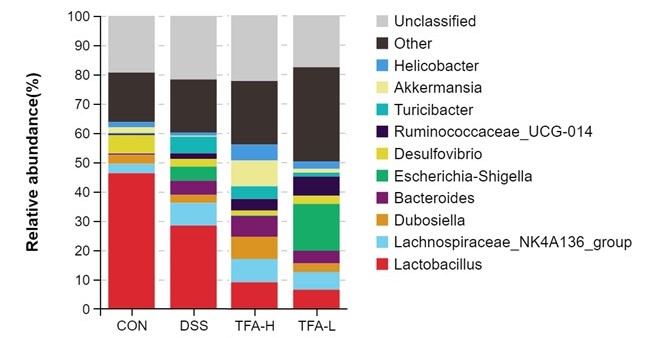
C.

Supplementary Figure 2 A. Heatmap showing the correlation between immune regulatory factors and gut microbiota. The spot with asterisk in red refers to the significant positive correlation (P < 0.05), and green indicates negative correlation (P < 0.05)

B. Heatmap of selected relative abundance greater than 0.1% at the genus level. The blue color represents less abundant, the red color represents the most abundant and white represents intermediate abundance.

C. Relative abundance of taxa at the genus levels.


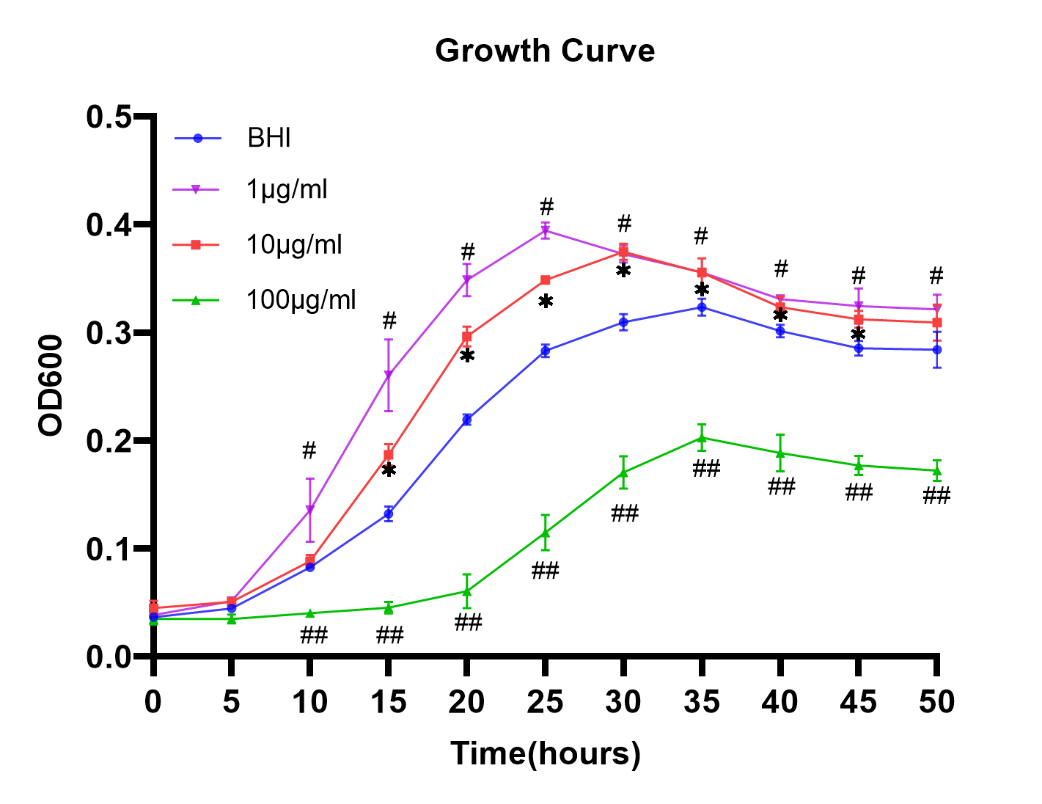
Supplementary Figure S3

Supplementary Figure S3. Growth curves of *Akkermansia muciniphila* under different conditions. Data are presented as the mean ± S.E.M. *P < 0.05, BHI compared with TFA (10µg/mL); #P < 0.05, BHI vs TFA(1µg/mL), ##P < 0.05, BHI vs TFA (100µg/mL)
